# Cerium Oxide Nanoparticles and Their Efficient Antibacterial Application *In Vitro* against Gram-Positive and Gram-Negative Pathogens

**DOI:** 10.3390/nano10081614

**Published:** 2020-08-18

**Authors:** Oana L. Pop, Amalia Mesaros, Dan C. Vodnar, Ramona Suharoschi, Flaviu Tăbăran, Lidia Magerușan, István Sz. Tódor, Zoriţa Diaconeasa, Adriana Balint, Lelia Ciontea, Carmen Socaciu

**Affiliations:** 1 Department of Food Science, University of Agricultural Science and Veterinary Medicine, 3-5 Calea Mănăştur Street, 400372 Cluj-Napoca, Romania; oana.pop@usamvcluj.ro (O.L.P.); dan.vodnar@usamvcluj.ro (D.C.V.); ramona.suharoschi@usamvcluj.ro (R.S.); zorita.sconta@usamvcluj.ro (Z.D.); carmen.socaciu@usamvcluj.ro (C.S.); 2 Physics and Chemistry Department, C4S Centre, Technical University of Cluj-Napoca, 28 Memorandumului Street, 400114 Cluj-Napoca, Romania; balintadriana@yahoo.com (A.B.); Lelia.Ciontea@chem.utcluj.ro (L.C.); 3 Department of Pathology, University of Agricultural Science and Veterinary Medicine, 3-5 Calea Mănăştur Street, 400372 Cluj-Napoca, Romania; flaviutabaran@gmail.com; 4 National Institute for Research and Development of Isotopic and Molecular Technologies, 65-103 Donath Street, 400293 Cluj-Napoca, Romania; lidia.magerusan@itim-cj.ro; 5 Faculty of Physics, Babeş-Bolyai University, 1st Kogălniceanu Street, 400084 Cluj-Napoca, Romania; istvan.todor88@gmail.com

**Keywords:** antibacterial activity, antioxidant activity, cerium oxide nanoparticles, foodborne pathogens

## Abstract

In this study, the antibacterial activity of cerium oxide nanoparticles on two Gram-negative and three Gram-positive foodborne pathogens was investigated. CeO_2_ nanoparticles (CeO_2_ nps) were synthesized by a Wet Chemical Synthesis route, using the precipitation method and the Simultaneous Addition of reactants (*WCS–SimAdd*). The as-obtained precursor powders were investigated by thermal analysis (TG–DTA), to study their decomposition process and to understand the CeO_2_ nps formation. The composition, structure, and morphology of the thermally treated sample were investigated by FTIR, Raman spectroscopy, X-ray diffraction, TEM, and DLS. The cubic structure and average particle size ranging between 5 and 15 nm were evidenced. Optical absorption measurements (UV–Vis) reveal that the band gap of CeO_2_ is 2.61 eV, which is smaller than the band gap of bulk ceria. The antioxidant effect of CeO_2_ nps was determined, and the antibacterial test was carried out both in liquid and on solid growth media against five pathogenic microorganisms, namely *Escherichia coli*, *Salmonella typhimurium*, *Listeria monocytogenes*, *Staphylococcus aureus*, and *Bacillus cereus*. Cerium oxide nanoparticles showed growth inhibition toward all five pathogens tested with notable results. This paper highlights the perspectives for the synthesis of CeO_2_ nps with controlled structural and morphological characteristics and enhanced antibacterial properties, using a versatile and low-cost chemical solution method.

## 1. Introduction

Antibiotics or antimicrobials have been widely utilized in various fields, such as medicine, food industry, agriculture, livestock, water treatment for diseases prevention, and pathogens eradication. Nevertheless, there is an increased concern related to multidrug-resistant microorganisms [1,2,3,4]. Worldwide, numerous scientists direct their efforts toward finding new alternatives for combating pathogens.

Among the many alternatives, such as plant extracts [5,6], antimicrobial peptides [7], and bee products [8,9], metal or metal oxide nanoparticles [10,11,12] are recently receiving increased attention. The properties found in the nanoparticle form of these materials can significantly differ from those of their bulk tantamount. Specific properties, such as considerably high contact surface and selectivity in the mediation of chemical transformations, facilitate their use in various areas.

Nanoparticles with various morphologies are utilized in multiple areas, including medicine, for the treatment or diagnosis [13,14,15,16,17,18] of pathogen-fighting systems [11,19] or improvement of the fuel quality [20,21].

Metal oxide nanoparticles, such as CeO_2_, have significant action on the antimicrobial activity and offer the plausibility of efficient pathogen removal from different environments. Furthermore, the stability and the slow release of metal ions from the nanoparticles are key features which make their usage advantageous. In comparison to other metal oxide nanoparticles, CeO_2_ has the quality of being an antioxidant due to the reversibility of its transfer from the reduced state into the oxidized state and resumes the process [22]. Its uses as an abrasive in semiconductor fabrication, or as a component in catalytic converters for auto machines exhaust systems, as a fuel additive to boost combustion, as an electrolyte for fuel, and as a UV-light absorber [21,23,24] are also well-known. Recently, nanoceria has been used as a colorimetric indicator for antioxidants activity due to the color changes induced by the modifications of cerium oxidation states at nanoparticle surface [25,26,27]. It was established that CeO_2_ nanoparticles have the capacity to initiate their mechanism by the modulation of the oxygen environment [28]. The properties of these CeO_2_ nanoparticles prove important possible biomedical applications. Scattered literature regarding the antibacterial activity of CeO_2_ nanoparticles is available. The effect against *E. coli* and *B. subtilis* [11], a comparison of the anti-bactericidal activity of bulk and nano CeO_2_ against the same Gram-negative bacteria [29,30], and cytotoxicity on nitrogen-fixing bacteria [31] are reported. Many studies have reported the synthesis of ceria nanoparticles (nps), using different cerium salts (acetate or nitrate) and different reactants (citric acid, ammonia, or hydrogen peroxide), and organic solvents/stabilizers (oleic acid, oleylamine, or diphenyl ether) to prevent particle agglomeration [32,33,34]. The addition of these organic species to the aqueous solution directly affects the stabilization level, the nucleation, and the growth processes of the CeO_2_ nps in the homogeneous solutions.

The aim of our work was the investigation of antibacterial properties of CeO_2_ nanoparticles against a wider range of pathogens, namely *Escherichia coli*, *Salmonella typhimurium*, *Listeria monocytogenes*, *Staphylococcus aureus*, and *Bacillus cereus*. A simple, reproducible, controllable, and low-cost wet-chemical synthesis method was used for the preparation of CeO_2_ nps. This precipitation-type method consists of the simultaneous addition of reactants (*SimAdd*) in the presence of an anti-agglomeration agent, under pH control, with the formation of cerium oxalate type salt as a precursor [35,36,37]. Based on the precursor characterization—thermal analysis and FTIR—the particle dimensionality and aggregation can be controlled. The CeO_2_ nps were characterized by using spectroscopic and microscopic methods, and their antioxidant and antibacterial activity was tested on two Gram-negative and three Gram-positive pathogens, respectively.

## 2. Materials and Methods

### 2.1. Materials

The chemicals used in the synthesis of the CeO_2_ nanoparticles are cerium nitrate hexahydrate, Ce(NO_3_)_3_∙6H_2_O (Sigma Aldrich, Chemie GmbH, Taufkirchen, Germany)—cation source, oxalic acid dihydrate, and H_2_C_2_O_4_∙2H_2_O (Alfa Aesar, Thermo Fisher Scientific Chemicals, Inc. Ward Hill, MA, USA)—precipitation agent, ammonia solution NH_4_OH, 30%NH_3_ (Alfa Aesar, Thermo Fisher Scientific Chemicals, Inc. Ward Hill, MA, USA) for pH control, tetraethylammonium hydroxide, (C_2_H_5_)_4_N(OH) as an anti-agglomeration agent, and NH_4_Cl (Alfa Aesar, Thermo Fisher Scientific Chemicals, Inc. Ward Hill, MA, USA) as flux. All chemicals were reagent grade and used without further purification.

### 2.2. Synthesis of Cerium Oxide Nanoparticles

The CeO_2_ nps samples were prepared via the Wet-Chemical Synthesis route, which consists of the Simultaneous Addition of reagents technique (*WCS–SimAdd*), using cerium nitrate and oxalic acid 50 mM aqueous solutions. The chemical reaction is presented in the Supplementary Materials file. The precipitation was carried out under continuous magnetic stirring, and the pH value was adjusted to 7 ± 0.2 by adding the ammonium hydroxide. Moreover, a small amount (1 vol%) of tetraethylammonium hydroxide solution was added to prevent the agglomeration of the particles. The post-precipitation stage consisted of 24 h aging, separation by filtering, and drying. The precursor nano-powders were intimately mixed with NH_4_Cl (flux), and the thermal treatment was performed at 400 °C/1 h, in air, at a heating rate of 300 °C/h. The thermally treated powders were washed several times with distilled water, until the filtrate was Cl^−^ free. Finally, the nano-powders were separated by centrifugation and dried in the oven at 110 °C for 1 h. A yellow aqueous solution (2 mM) was obtained by dispersing a specific mass of ceria nps in distilled water.

### 2.3. Characterization of Cerium Oxide Nanoparticles

The thermal decomposition process of cerium oxalate–type precursor was monitored by thermal analysis, using a Mettler Toledo TGA/SDTA851 (Greifensee, Switzerland) system, a platinum crucible, and a heating rate of 10 K/min, in a static air atmosphere up to 500 °C. The chemical nature of the precursor and final oxide was analyzed by Fourier Transform Infrared Spectroscopy (FTIR), using a Tensor 27 Bruker FTIR spectrophotometer (Bruker Optik GmbH, Germany). The crystalline structure of the CeO_2_ nano-powders was determined by X-ray diffraction (XRD) investigations, which were performed at room temperature, by means of a Bruker AXS D8 Discover diffractometer (Bruker, Karlsruhe, Germany) operating at 40 kV, 40 mA, CuK_α_ radiation λ = 1.54056 Å. The hydrodynamic diameter was determined by dynamic light scattering (DLS) measurements, using a Brookhaven Instruments Corporation (Holtsville, NY, USA), a goniometer, and a laser light-scattering system. The acquisition time was set to 90 s, a laser radiation wavelength of 632.8 nm was used, and the angle at which data acquisition was performed was 90°. During long-term storage, the solution gradually lost its stability due to the formation of nanoparticle aggregates. TEM was utilized to characterize the morphology of CeO_2_ nps. A JEOL JEM1010 transmission electron microscope (JEOL USA, Inc., Peabody, MA, USA) was used, operating at an accelerating voltage of 100 kV and equipped with a MegaViewIII CCD camera. The optical characterization was performed by a Jasco V 530 spectrophotometer (Jasco Corporation, Tokyo, Japan), in the range of 200–800 nm. The Raman spectra of the CeO_2_ nanoparticles were recorded at room temperature, using a Renishaw in Via Reflex Raman Microscope (New Mills, UK) equipped with a RenCam CCD detector. The Raman system was operated with 532 nm laser line, and the spectra were recorded by adding 4 accumulations, each with a 40 s exposure time, acquired by using 13.5 mW laser power with a 4 cm^−1^ resolution.

### 2.4. Antioxidant Properties of CeO_2_ Nps SCAVENGING Effect on ABTS Radicals

The scavenging properties of CeO_2_ nps against radical cations ABTS^+^ (2,2’-azino-bis(3-ethylbenzothiazoline-6-sulfonic acid)) were determined according to the procedure described in other studies [38,39], adapted to a microplate of 96 wells. The ABTS assay is based on the capacity of a sample to scavenge the ABTS radical cation (ABTS. ABTS ^+^), as compared to a standard antioxidant (Trolox). The blue–green ABTS^+^ solution was produced by the reaction between a 7 mM aqueous solution of ABTS and a 2.45 mM potassium persulfate, in a dark medium, and at room temperature, for 12–16 h before use. ABTS^+^ working solution was obtained by diluting the stock solution with ethanol, resulting in an absorbance of 0.70 ± 0.02 AU at 734 nm. Then, 20 µL of Trolox or CeO_2_ nps solution at different concentrations were added to 170 µL ABTS^+^ solutions, and the absorbance was measured after 6 min of incubation, in the dark, and at room temperature, using a microplate reader. The results were expressed as µmol Trolox per Gram sample.

### 2.5. Antibacterial Activity

The antibacterial activity of the tested nanoparticles was evaluated by using different methods, such as disk diffusion tests, UV–Vis measurements of the optical density (OD), the number of colony-forming units (CFUs) on solid medium, the resazurin test, and bacterial viability, using confocal microscopy. The five pathogenic strains used in the study were *Escherichia coli* ATC 25922 (Microbiologics), *Salmonella typhimurium* ATCC 14028 (Microbiologics) *Listeria monocytogenes* ATCC 35152 (Liofilclem), *Staphylococcus aureus* ATCC 65389 (MediTech), and *Bacillus cereus* ATCC 11778 (MediTech).

*Disk diffusion method*—About 15 mL of sterile nutrient agar (Bioaqua, Targu-Mures) was poured into the sterile Petri dish. Triplicate plates were inoculated with 200 µL of the overnight culture (approximately 10^8^ CFU/mL) of the targeted pathogenic bacteria, which were spread on the plate, using sterile Drigalski spatulas. On the solid medium were gently placed up to 6 paper disks that were soaked with a fixed concentration of CeO_2_ nps solution. The plates were incubated for 24 h at 37 ± 1 °C, prior to the determination of results. The zone of inhibition around each of the paper disks was measured and expressed as millimeters in diameter. Gentamicin was used as the control.

*Minimum Inhibitory Concentration (MIC)*—Broth dilution tests were conducted in standard trays containing 96 wells, to evaluate the MIC of the cerium oxide nanoparticles. To obtain sequential dilutions, 150 µL of the cerium oxide nanoparticles was homogenized with 150 µL of nutrient broth; afterward, the solution was well mixed, and then 150 µL of the mixture was transferred in the next well containing 150 µL of nutrient broth. The final volume was of 200 µL in each well after the addition of 50 µL of 24-hour-old bacteria inoculum. The final solution was allowed to grow at 37 °C for 24 h. Growth curves for the control of pathogenic cells incubated with three different concentrations of CeO_2_ nps were measured at OD550. The MIC value was the lowest concentration of the nanoparticles that did not permit any visible growth of the pathogenic bacteria during 24 h of incubation because of turbidity. The negative control was the sample where no bacteria were added, and the positive control was the sample where no cerium oxide nanoparticles were added. The turbidity was measured by using UV–Vis spectroscopy (microplate reader (HT BioTek Synergy) at 550 nm. The same experiment was conducted, using gentamicin as an antibiotic. All the experiments were performed in triplicates and were repeated twice.

*Minimum Bactericidal Concentration (MBC)*—To prevent the possibility of misinterpretation due to the turbidity of insoluble compounds, the MBC was established by sub-culturing the above (MIC) serial dilutions after the incubation time in nutrient agar plates, using a 0.01 mL loop, and further incubating the dilutions at 37 °C for 24 h. The MBC was considered to be the lowest concentration that prevents the growth of the bacterial colony on this solid medium.

*Bacterial viability using confocal microscopy*—The confocal laser scanning microscope - Zeiss LSM 710 (Oberkochen, Germany) - is a method that allowed us to view live and dead bacteria. Bacteria cells culture (~10^8^ CFU) were treated with 50 µg/mL of CeO_2_ nps for 24 h. A control with no treatment was made for each pathogen. Prior to the confocal microscopy assay, the bacteria cells were mixed, for coloration, with a LIVE/DEAD^®^ BacLight™ bacterial viability Kit, L-7007, for 15 min, in the dark. Aliquots of 50 µL of the colored bacteria were placed on a microscope slide, fixed using a flame, rinsed, and dried.

*Time kill assay*—The potential CeO_2_ nanoparticle was subject to the time killing assay. An inoculum of the tested pathogens (10 µL), at a concentration of 10^8^ CFU/mL, was mixed with 100 µL of solution containing growth medium and nanoparticles, to a final concentration of 50 µg/mL. The negative controls were the samples in which no nanoparticles were utilized. The growth of bacterial species was assessed at every 1 h interval, by measuring the optical density at 550 nm with the microplate reader.

## 3. Results and Discussion

**Figure 1:**
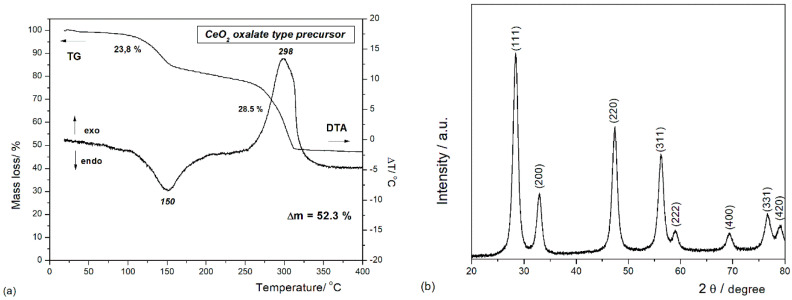
(**a**) Thermal analysis of the cerium oxalate precursor (total theoretical weight loss is 52.49%). (**b**) XRD pattern recorded at room temperature for CeO_2_ nanoparticles (nps).

It is well-known that the critical step in the synthesis of metal oxide by wet-chemical methods is the thermal decomposition of the obtained precursor. The thermal analysis method (DTA–TG) allowed us to investigate the chemical transformation of the oxalate-type precursor into oxide powders during heating (see Figure 1a). The thermal behavior of the synthetized precursor through simultaneous DTA–TG measurements reveals two weight-loss processes. In the low temperature range, the DTA curve illustrates an endothermic process with the maximum temperature centered around 150 °C, accompanied by a mass loss of 23.8% on the TG curve. This first decomposition step is attributed to the dehydration of the precursor with the loss of around 10 water molecules and the formation of anhydrous cerium oxalate [40,41,42]. The second weight loss (~28.5%) is observed at temperatures ranging between 250 and 350 °C and corresponds to the oxidative decomposition of the oxalate group, as indicated by the exothermic effect, whose maxima is located at 298 °C. Almost no weight loss is observed at temperatures higher than 350 °C in the TG curve, indicating that the oxalate precursor was completely decomposed with the formation of CeO_2_. This decomposition behavior confirms that the chemical composition of the oxalate precursor is close to the molecular formula of Ce_2_(C_2_O_4_)_3_∙10H_2_O, which is in concordance with the literature data [40,41,43]. Based on these observations, one can assume that the precursor decomposition takes place according to the following chemical reactions:

100–200 °C: Ce_2_(C_2_O_4_)_3_·10H_2_O → Ce_2_(C_2_O_4_)_3_ + 10H_2_O

250–350 °C: Ce_2_(C_2_O_4_)_3_ → 2CeO_2_ + 2CO_2_ + 4CO

To identify the crystalline phases and to estimate the average particle sizes, an XRD investigation was performed. The XRD pattern of the oxide powders obtained after annealing at 400 °C for 1 h is presented in Figure 1b. All the reflections were indexed with those of the pure cubic fluorite structure of CeO_2_ (JCDD PDF 034-0394) [30,33,40]. The average crystallite sizes of the sample were calculated by using the Debye–Scherrer formula:

(1) $$D_{p}=\frac{0.9\cdot \lambda }{\beta \cdot cos\theta }$$

where *D_p_* is the average crystallite size, *λ* is the wavelength of the CuKα line, *θ* is the Bragg angle, and *β* is the Full Width at Half-Maximum (FWHM) of the diffraction peak in radians. The average crystallite size calculated for (111), (200), (220), and (311) reflections was about 7 nm.

**Figure 2:**
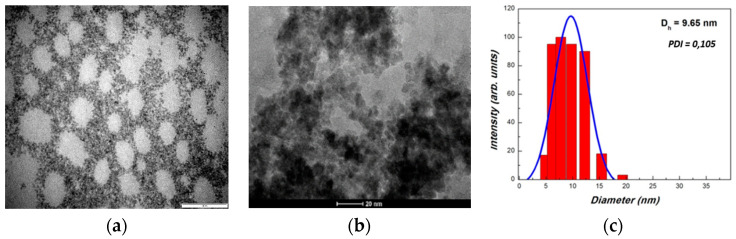
TEM and HRTEM images (**a**,**b**) and DLS measurement (**c**) of thermally treated CeO_2_ nps. The scales are 1 μm (**a**) and 20 nm (**b**).

To further analyze the morphology—size and shape—of CeO_2_ nps, TEM–HRTEM and DLS characterizations were carried out, and the results are presented in Figure 2a–c. The TEM images illustrate the presence of aggregates with pseudo-spherical shapes. This behavior to form larger and denser nanoparticle aggregates during the thermal treatment is characteristic for oxalate-type precursors [42]. DLS measurements of the CeO_2_ nps suspensions present an average hydrodynamic diameter (D_h_) of 9.65 nm, with the size of the nanoparticles averaging in the range of 5–20 nm. This larger value for D_h_ is related to the interference of the dispersant into the hydrodynamic diameter.

**Figure 3:**
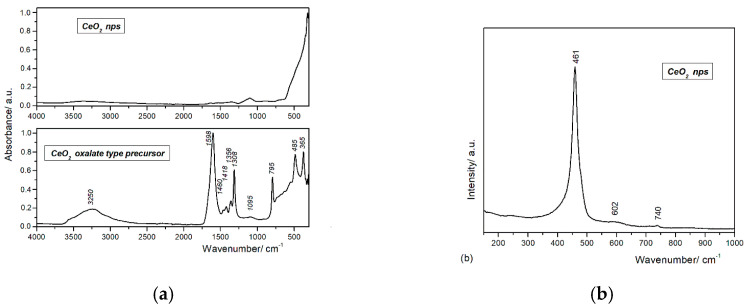
(**a**) FTIR spectra of cerium oxalate–type precursor and CeO_2_ nps (4000–350 cm^−1^). (**b**) Raman spectrum of CeO_2_ nps.

Figure 3a shows the FTIR spectra of the synthetized CeO_2_ nps and the cerium oxalate–type precursor. The large band observed in the 3500–3000 cm^−1^ domain can be assigned to the collective in-phase symmetric ν(O–H) stretching vibration [44]. The asymmetric ν_asym_(COO^−^) observed at 1598 cm^−1^ and the symmetric ν_sym_(COO^−^) stretching modes observed 1356 and 1308 cm^−1^ are assigned to the oxalate modes [45]. The difference in their frequencies, Δν(COO^−^) = ν_asym_(COO^−^)—ν_sym_(COO^−^), is around 298 cm^−1^ and is indicative of the formation of a bidentate coordination bond between the cerium ions and the oxalate group. Moreover, the low-intensity vibration bands at 1460 and 1418 cm^−1^ suggest the existence of both mononuclear and binuclear bidentate oxalate complexes [45,46]. The low intensity band observed at 1095 cm^−1^ is characteristic for ν_as_(C–O–C) and ν(Ce–O–C) vibration [45,47]. The bands at 795, 485, and 365 cm^−1^ are attributed to the δ(O–C=O) bridging oxalate group and to the interaction of the metal–oxygen vibration ν(Ce–O) [45,46,47,48]. The FTIR spectrum of CeO_2_ nps confirms the total conversion of the oxalate precursor into ceria by thermal treatment. The presence of the absorption bands characteristic to Ce–O vibrations sustains the main phase of the as-prepared particles as cerium oxide, and the thermal treatment assures the complete decomposition of the oxalate-type precursor.

Raman spectroscopy is one of the major tools utilized in the study of crystallinity, purity, and any defect levels connected to the nanomaterials. A typical Raman spectrum for bulk CeO_2_ with cubic fluorite structure presents a single strong peak at 464 cm^−1^, assigned to the F_2g_ symmetry modes of CeO_2_ [49,50]. The Raman spectrum of the synthetized CeO_2_ nps (see Figure 3b) exhibits a strong Raman absorption peak at 461 cm^−1^ and two weak absorption peaks at 610 and 740 cm^−1^. The Raman peak at 461 cm^−1^ corresponds to the triply degenerate F_2g_ mode of symmetric stretching vibration of oxygen ions around Ce^4+^ cations and therefore confirms the cubic fluorite structure. The slight broadness of the peak is attributed to the reduction of the particle size to nanometric ranges. The shift in the peak position toward the lower energy value and the broad peaks at 610 and 740 cm^−1^ are due to the presence of point defects generated by oxygen vacancies [49,50,51].

**Figure 4:**
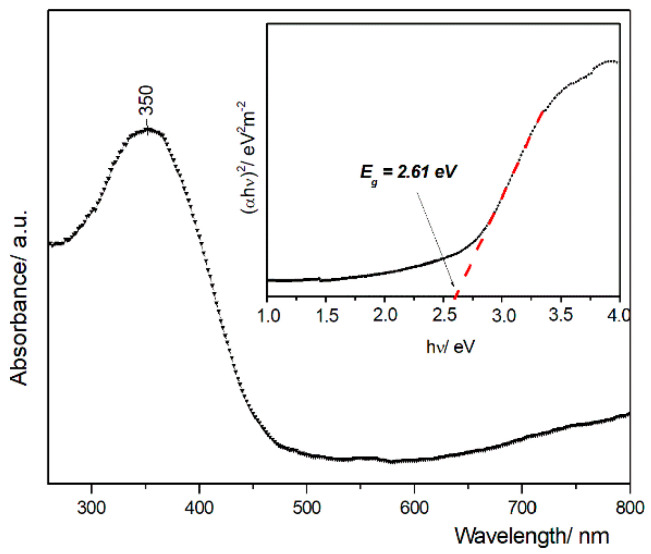
UV–Vis diffuse absorption spectra of the obtained CeO_2_ nps and optical band gap determination from the plot of (αhν)^2^ versus photon energy (αh)—the inset.

The UV–Vis spectrum is shown in Figure 4. The maximum absorption peak at 350 nm is assigned to the charge transfer between the O 2p and Ce 4f states in O^2−^ and Ce^4+^. Compared with CeO_2_ bulk, where the absorption peak is around 389 nm, the maximum intensity for the synthetized CeO_2_ nps is shifted toward a lower wavelength value (blueshift) [50]. Based on the absorption spectra, the direct band gap of CeO_2_ nps was determined from the following relationship:

*αhν* = *A* (*hν* − *E_g_*)^*n*/2^ (2)

where *α* is the optical absorption coefficient, *α* = *A/d^’^*; *A*—is the measured absorbance, and *d^’^* is the thickness of sample in a UV–Vis cell (0.4 cm); *hν* is the photon energy; and *E_g_* is the direct band gap of the sample. The extrapolation of the linear part of the domain of (*αhν*)^2^ vs. *hν* to the *αhν* = 0 (where *hν = E_g_*) allows the determination of the direct band gap of E_g_ value (2.61 eV). The red shift in the band gap is associated with oxygen vacancies and existence of Ce^3+^ to the nanoparticle surface. Oxygen defects and Ce^3+^ ions generate intermediate defect energy states in the CeO_2_ band gap. Due to the presence of these states, the direct transition of electrons from O 2p to Ce 4f is slowed down, resulting in the decrease of band gap value [50,52,53].

**Figure 5:**
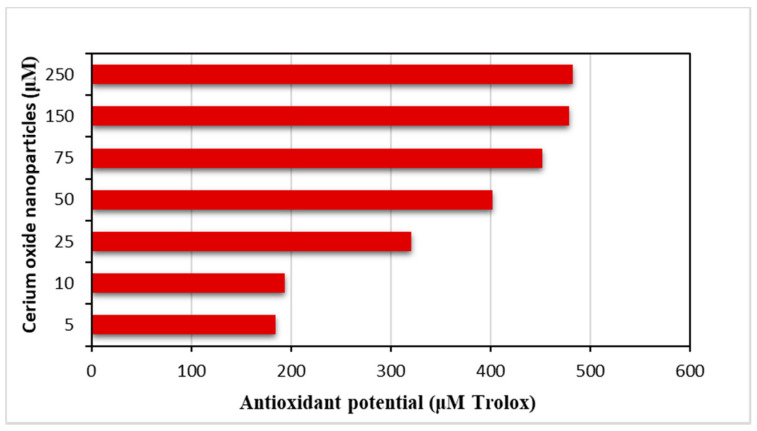
Antioxidant capacity of CeO_2_ nps estimated by ABTS•−assays (expressed as μmol trolox /g sample).

To evaluate the antioxidant potential of CeO_2_ nps, the ABTS (2,2’-azino-bis(3-ethylbenzthiazoline)-6-sulphonic acid) assay was tested. The ABTS method is indicated for a suitable and continuous spectrophotometric measurement of any potential radical formations. ABTS creates a characteristic cation radical (ABTS^+^) that can be easily followed and can allow the measurement of the initial rate of radical formation. The obtained results (see Figure 5 and Table S1 in Supplementary Materials file) have clearly demonstrated that the CeO_2_ nps is a radical scavenger and can inhibit the ABTS^+^ radical formation in a dose-dependent manner [54]. The results were expressed as Trolox equivalents. Trolox is a water-soluble analogue of vitamin E. Trolox is an antioxidant that has been shown to prevent lipid peroxidation in vivo. Previous publications have shown that vitamin E is effective in preventing oxidative harm from iron and other redox-active metals in vivo and in vitro, likely by reacting with hydroxyl radicals or downstream radical intermediates [55,56]. It can be easily concluded that the antioxidant activity increases in direct proportionality with the nanoparticle concentration.

Compared with bulk, CeO_2_ nps exhibit superior properties due to their small size, which allows a rapid adsorption of pathogens. Many studies have shown that the CeO_2_ nps exhibit excellent antibacterial activity against Gram-positive and Gram-negative bacteria due to the generation of reactive oxygen species (ROS) [57,58,59]. In healthy cells, CeO_2_ nps act as antioxidants by scavenging ROS at a physiological pH, while, in pathogens—under low-pH environment—CeO_2_ nps act as a pro-oxidant by generating ROS and producing cell damage. Zhang et al. [59] presented a systematic research on the antibacterial mechanism and further summarized the main steps and influencing factors of these reactions.

The antibacterial activity of CeO_2_ nps was evaluated by using the microdilution method, and the corresponding MIC and MBC values were evaluated against two Gram-negative and three Gram-positive pathogens. The correspondent values are given in Table 1 and Table 2, respectively. The cerium oxide nanoparticles demonstrated antibacterial properties against all the tested pathogens in relative low concentrations.

The antibacterial activity of CeO_2_ nps discloses that the same concentration of nanoparticles against *Escherichia coli* and *Salmonella typhimurium* resulted in different diameters of inhibition, with these being 9 and 10 mm, respectively. Regarding the Gram-positive pathogens, the highest inhibition was registered in the case of *Listeria monocytogenes*, followed by *Bacillus cereus* and *Staphylococcus aureus*. The MBC results reveal that the highest sensitivity of solely 1.07 g/L CeO_2_ nps can be observed in *Salmonella typhimurium* and *Listeria monocytogenes*. The difference in the action of CeO_2_ nps against the Gram-positive and Gram-negative pathogens is primarily related to the different structure and compactness of their cell walls. Usually, Gram-positive bacteria have a thicker, waxy cell wall, making them more resistant to the antimicrobial activity of CeO_2_ nps in comparison with Gram-negative bacteria [60,61]. For example, the Gram-positive *Bacillus cereus* has a cell wall of 55.4 nm, while the Gram-negative *S. typhimurium* has a cell wall of only 2.4 nm [62]. Even if important functional differences can be seen between the Gram-positive and Gram-negative bacteria cell wall, in the DNA-based molecular taxonomy, some pathogens have a similar response to the same antibacterial agents [63].

**Figure 6:**
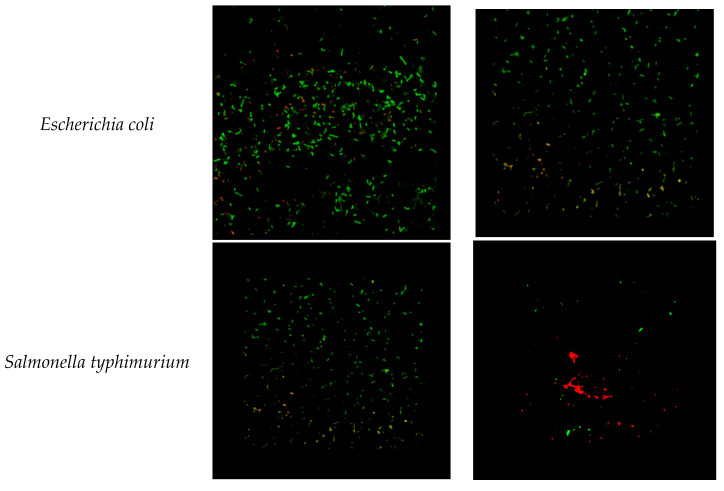

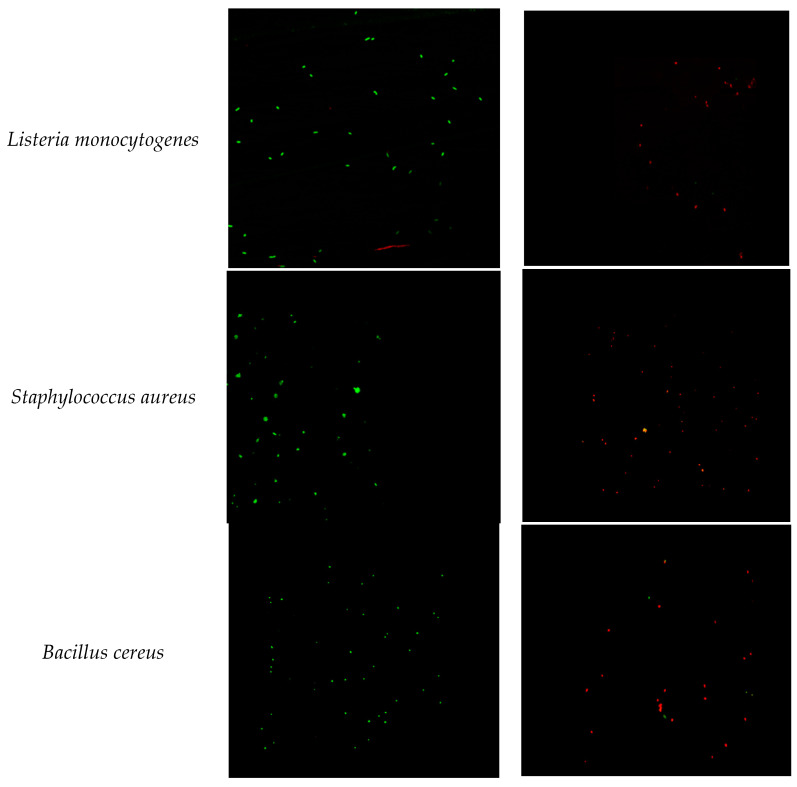
LIVE/DEAD^®^ BacLight™ bacterial viability kit control (**left**) and after 24 h of treatment with 50 µg/mL CeO_2_ nps (**right**): *Escherichia coli*, *Salmonella typhimurium*, *Listeria monocytogenes*, *Staphylococcus aureus*, and *Bacillus cereus*. Live cells (expressing SYTO^®^ 9) are shown in green, and dead cells (expressing propidium iodide) are shown in red. (Original magnification: × 60).

The antibacterial effect of CeO_2_ nps is confirmed by the confocal microscopy images from Figure 6. The results clearly evidence that the inhibitory effect of CeO_2_ nps is present at a lower concentration, with respect to gentamicin (the standard drug). This fact guarantees the presence of the antibacterial action of CeO_2_ nps against the tested pathogens.

**Figure 7:**
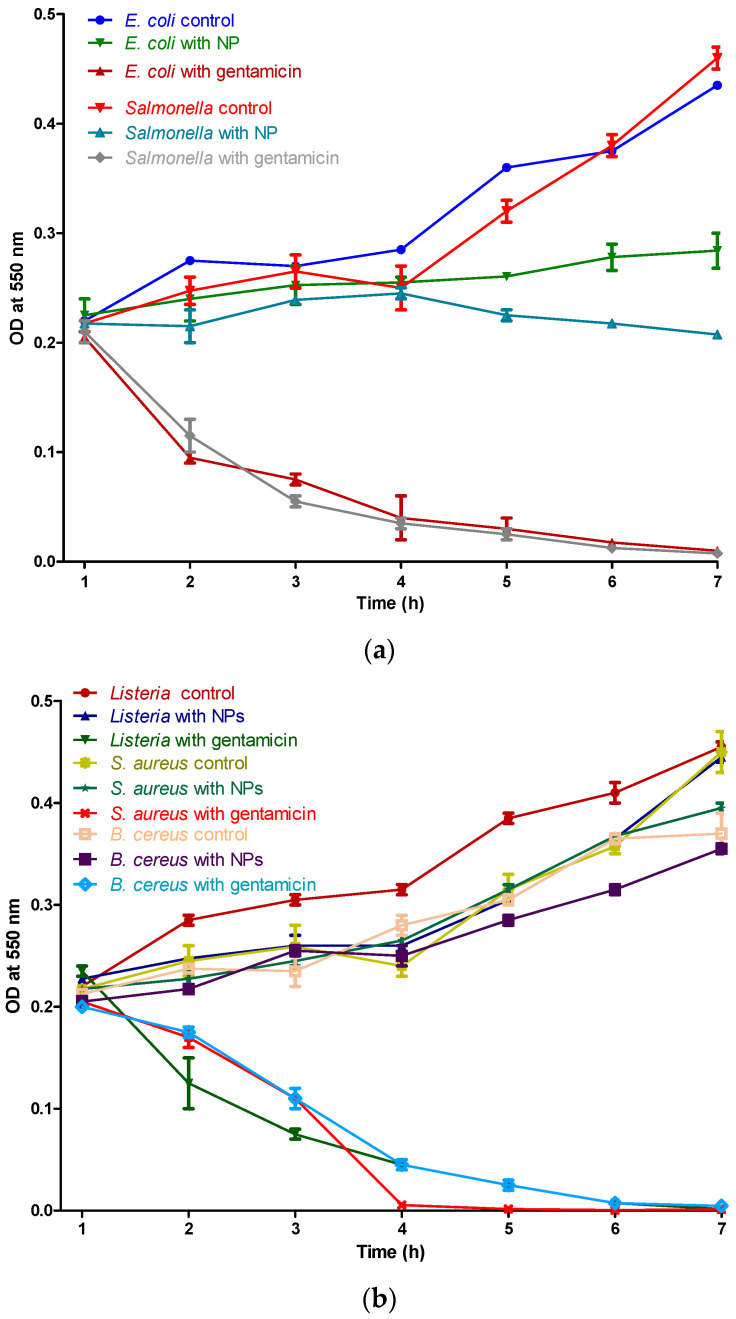
Time kill assay of CeO_2_ nps against the Gram-negative bacteria (*E. coli* and Salmonella) (**a**) and Gram-positive bacteria (Listeria, *S. aureus*, and *B. cereus*) (**b**).

The time kill assay shows an inhibitory effect in a time-dependent manner, in Figure 7, indicating that CeO_2_ nps interaction with the tested pathogens results in cell damage. It can be observed that the bacterial growth was inhibited from the first hour.

A feasible mechanism of action can be explained by the fact that the CeO_2_ nps are carrying positive charges, and the bacteria are charged negatively, inducing electromagnetic attraction. This attraction ensures direct contact between nanoparticles and bacteria, leading to oxidation and bacterial cellular death [11].

## 4. Conclusions

Cerium oxide nanoparticles have been designed through a wet chemical method by using the simultaneous addition of reactants (*WCS–SimAdd*). A better understanding of the decomposition mechanism for as-obtained oxalate precipitate was achieved by DTA–TG and FTIR analyses. The cubic fluorite structure of the cerium oxide nps was ascertained by XRD and Raman spectroscopy. TEM and DLS revealed the morphology, showing that the sizes are in the range of 5–20 nm, with pseudo-spherical shapes and a lower tendency to form aggregates. The oxygen vacancies and formation of Ce^3+^ evidenced by Raman and UV–Vis spectroscopies are correlated to the particle low-dimensionality.

Experiments prove that the synthetized CeO_2_ nps exhibit excellent antibacterial activity against the five tested bacterial species: *E. coli*, *S. typhimurium*, *L. monocytogenes*, *S. aureus*, and *B. cereus*. Low MIC values were registered, a result that suggests the suitable antibacterial characteristics of CeO_2_ nps. Antibacterial action is attributable to the direct interaction between the cerium oxide nanoparticles and the bacteria which induces the cellular death of these pathogens. In overview, this study evidenced the potential antibacterial property of cerium oxide nanoparticles, a fact that leads to new approaches in the development of biomedical and food applications. Further studies, such as long-term treatment, are important for a better understanding and the application of CeO_2_ nps in various antibacterial applications.

## Figures and Tables

**Table 1:** Antibacterial activity of CeO_2_ nps at 50 µg/mL and gentamicin 10 µg/mL, against pathogenic bacteria.

	Pathogenic Bacteria	CeO_2_ nps Zone of Inhibition (mm Diameter)	Gentamicin Zone of Inhibition (mm Diameter)
G -	*Escherichia coli*	9 ± 0.05	15 ± 0.43
	*Salmonella typhimurium*	12 ± 0.02	17 ± 0.02
G +	*Listeria monocytogenes*	10 ± 0.04	19 ± 0.01
	*Staphylococcus aureus*	5 ± 0.02	18 ± 0.02
	*Bacillus cereus*	7 ± 0.05	16 ± 0.01

**Table 2:** Minimum Inhibitory Concentration (MIC) and Minimum Bactericidal Concentration (MBC) of CeO_2_ nps/gentamicin against pathogenic bacteria. Experiments were performed in triplicates and were repeated twice.

Bacterial Strains	CeO_2_ nps MIC (mg/mL)	Gentamicin MIC (mg/mL)	CeO_2_ nps MBC (mg/mL)
*Escherichia coli*	2.15	3.0	2.15
*Salmonella typhimurium*	1.07	0.38	1.07
*Listeria monocytogenes*	1.07	1.2	1.07
*Staphylococcus aureus*	10	0.83	10
*Bacillus cereus*	4.3	0.39	4.3

## Supplementary Materials

The following are available online at https://www.mdpi.com/2079-4991/10/8/1614/s1, Table S1: The antioxidant activity data.

## References

[1] Allen H.K., Levine U.Y., Looft T., Bandrick M., Casey T.A. (2013). Treatment, promotion, commotion: Antibiotic alternatives in food-producing animals. Trends Microbiol..

[2] Defoirdt T., Sorgeloos P., Bossier P. (2011). Alternatives to antibiotics for the control of bacterial disease in aquaculture. Curr. Opin. Microbiol..

[3] Nigam A., Gupta D., Sharma A. (2014). Treatment of infectious disease: Beyond antibiotics. Microbiol. Res..

[4] Wilson H.L., Buchanan R.M., Allan B., Tikoo S.K. (2012). Milk-derived antimicrobial peptides to protect against neonatal diarrheal disease: An alternative to antibiotics. Procedia Vaccinol..

[5] Diaconeasa Z., Ranga F., Rugina D., Leopold L., Pop O.L., Vodnar D.C., Lucian C., Carmen S. (2015). Phenolic content and their antioxidant activity in various berries cultivated in Romania. Bull. UASVM Food Sci. Technol..

[6] Gyawali R., Hayek S.A., Ibrahim S.A., Taylor T.M. (2014). Plant extracts as antimicrobials in food products: Mechanisms of action, extraction methods, and applications. Handbook of Natural Antimicrobials for Food Safety and Quality.

[7] De Freitas Lima S.M., de Pádua G.M., da Costa Sousa M.G., de Souza Freire M., Franco O.L., Rezende T.M.B. (2015). Antimicrobial peptide-based treatment for endodontic infections—Biotechnological innovation in endodontics. Biotechnol. Adv..

[8] Mihai C.M., Mărghitaş L.A., Dezmirean D.S., Chirilă F., Moritz R.F., Schlüns H. (2012). Interactions among flavonoids of propolis affect antibacterial activity against the honeybee pathogen Paenibacillus larvae. J. Invertebr. Pathol..

[9] Urcan A., Criste A., Dezmirean D., Bobiș O., Marghitas L., Margaoan R., Hrinca A. (2018). Antimicrobial Activity of Bee Bread Extracts Against Different Bacterial Strains. Bull. Univ. Agric. Sci. Vet. Med. Cluj-Napoca. Anim. Sci. Biotechnol..

[10] Dizaj S.M., Lotfipour F., Barzegar-Jalali M., Zarrintan M.H., Adibkia K. (2014). Antimicrobial activity of the metals and metal oxide nanoparticles. Mater. Sci. Eng. C.

[11] Pelletier D.A., Suresh A.K., Holton G.A., McKeown C.K., Wang W., Gu B., Mortensen N.P., Allison D.P., Joy D.C., Allison M.R. (2010). Effects of engineered cerium oxide nanoparticles on bacterial growth and viability. Appl. Environ. Microbiol..

[12] Dahle J.T., Arai Y. (2015). Environmental geochemistry of cerium: Applications and toxicology of cerium oxide nanoparticles. Int. J. Environ. Res. Public Health.

[13] Bridle H. (2014). Nanotechnology for Detection of Waterborne Pathogens. Waterborne Pathogens.

[14] Chigurupati S., Mughal M.R., Okun E., Das S., Kumar A., McCaffery M., Seal S., Mattson M.P. (2013). Effects of cerium oxide nanoparticles on the growth of keratinocytes, fibroblasts and vascular endothelial cells in cutaneous wound healing. Biomaterials.

[15] Choi H., Lee K., Hur N., Lim H. (2014). Cerium oxide-deposited mesoporous silica nanoparticles for the determination of carcinoembryonic antigen in serum using inductively coupled plasma-mass spectrometry. Anal. Chim. Acta.

[16] Coman C., Leopold L.F., Rugină O.D., Barbu-Tudoran L., Leopold N., Tofană M., Socaciu C. (2014). Green synthesis of gold nanoparticles by Allium sativum extract and their assessment as SERS substrate. J. Nanoparticle Res..

[17] Wason M.S., Colon J., Das S., Seal S., Turkson J., Zhao J., Baker C.H. (2013). Sensitization of pancreatic cancer cells to radiation by cerium oxide nanoparticle-induced ROS production. Nanomed. Nanotechnol. Biol. Med..

[18] Hosseini M., Mozafari M. (2020). Cerium oxide nanoparticles: Recent advances in tissue engineering. Materials.

[19] Azam A., Ahmed A.S., Oves M., Khan M.S., Habib S.S., Memic A. (2012). Antimicrobial activity of metal oxide nanoparticles against Gram-positive and Gram-negative bacteria: A comparative study. Int. J. Nanomed..

[20] Mori T., Ou D.R., Zou J., Drennan J. (2012). Present status and future prospect of design of Pt–cerium oxide electrodes for fuel cell applications. Prog. Nat. Sci. Mater. Int..

[21] Selvan V.A.M., Anand R., Udayakumar M. (2014). Effect of Cerium Oxide Nanoparticles and Carbon Nanotubes as fuel-borne additives in Diesterol blends on the performance, combustion and emission characteristics of a variable compression ratio engine. Fuel.

[22] Corral-Diaz B., Peralta-Videa J.R., Alvarez-Parrilla E., Rodrigo-García J., Morales M.I., Osuna-Avila P., Niu G., Hernandez-Viezcas J.A., Gardea-Torresdey J.L. (2014). Cerium oxide nanoparticles alter the antioxidant capacity but do not impact tuber ionome in Raphanus sativus (L). Plant Physiol. Biochem..

[23] Mos R., Petrisor T., Nasui M., Calleja A., Puig T., Ciontea L., Petrisor T. (2014). Enhanced structural and morphological properties of Gd-doped CeO_2_ thin films obtained by polymer-assisted deposition. Mater. Lett..

[24] Snow S.J., McGee J., Miller D.B., Bass V., Schladweiler M.C., Thomas R.F., Krantz T., King C., Ledbetter A.D., Richards J. (2014). Inhaled diesel emissions generated with cerium oxide nanoparticle fuel additive induce adverse pulmonary and systemic effects. Toxicol. Sci..

[25] Andrei V., Sharpe E., Vasilescu A., Andreescu S. (2016). A single use electrochemical sensor based on biomimetic nanoceria for the detection of wine antioxidants. Talanta.

[26] Karimi A., Othman A., Andreescu S. (2016). Portable enzyme-paper biosensors based on redox-active CeO_2_ nanoparticles. Methods in Enzymology.

[27] Othman A., Norton L., Finny A.S., Andreescu S. (2020). Easy-to-use and inexpensive sensors for assessing the quality and traceability of cosmetic antioxidants. Talanta.

[28] Das S., Singh S., Dowding J.M., Oommen S., Kumar A., Sayle T.X., Saraf S., Patra C.R., Vlahakis N.E., Sayle D.C. (2012). The induction of angiogenesis by cerium oxide nanoparticles through the modulation of oxygen in intracellular environments. Biomaterials.

[29] Kuang Y., He X., Zhang Z., Li Y., Zhang H., Ma Y., Wu Z., Chai Z. (2011). Comparison study on the antibacterial activity of nano-or bulk-cerium oxide. J. Nanosci. Nanotechnol..

[30] Krishnan A., Sreeremya T.S., Murray E., Ghosh S. (2013). One-pot synthesis of ultra-small cerium oxide nanodots exhibiting multi-colored fluorescence. J. Colloid Interface Sci..

[31] Bandyopadhyay S., Peralta-Videa J.R., Plascencia-Villa G., José-Yacamán M., Gardea-Torresdey J.L. (2012). Comparative toxicity assessment of CeO_2_ and ZnO nanoparticles towards Sinorhizobium meliloti, a symbiotic alfalfa associated bacterium: Use of advanced microscopic and spectroscopic techniques. J. Hazard. Mater..

[32] Babenko L., Zholobak N., Shcherbakov A., Voychuk S., Lazarenko L., Spivak M.Y. (2012). Antibacterial activity of cerium colloids against opportunistic microorganisms in vitro. Microbiol. J..

[33] Chen H.-I., Chang H.-Y. (2005). Synthesis of nanocrystalline cerium oxide particles by the precipitation method. Ceram. Int..

[34] Ivanova O.S., Shekunova T.O., Ivanov V.K., Shcherbakov A.B., Popov A.L., Davydova G.A., Selezneva I.I., Kopitsa G.P., Tret’yakov Y.D. (2011). One-stage synthesis of ceria colloid solutions for biomedical use. Dokl. Chem..

[35] Mesaros A., Ghitulica C.D., Popa M., Mereu R., Popa A., Petrisor T., Gabor M., Cadis A.I., Vasile B.S. (2014). Synthesis, structural and morphological characteristics, magnetic and optical properties of Co doped ZnO nanoparticles. Ceram. Int..

[36] Muresan L., Popovici E., Indrea E. (2011). Structural and luminescence characterization of yttrium oxide based phosphors prepared by wet-chemical method. J. Optoelectron. Adv. Mater..

[37] Marius M., Popovici E.J., Barbu-Tudoran L., Indrea E., Mesaros A. (2014). Cerium-doped yttrium aluminate-based phosphors prepared by wet-chemical synthesis route: Modulation of the luminescence color by changing the host-lattice composition. Ceram. Int..

[38] Arnao M.B., Cano A., Acosta M. (2001). The hydrophilic and lipophilic contribution to total antioxidant activity. Food Chem..

[39] Zhao L., Peralta-Videa J.R., Peng B., Bandyopadhyay S., Corral-Diaz B., Osuna-Avila P., Montes M.O., Keller A.A., Niu G., Gardea-Torresdey J.L. (2014). Alginate modifies the physiological impact of CeO_2_ nanoparticles in corn seedlings cultivated in soil. J. Environ. Sci..

[40] Gabal M., Elroby S.A., Obaid A. (2012). Synthesis and characterization of nano-sized ceria powder via oxalate decomposition route. Powder Technol..

[41] Maslennikov D., Matvienko A., Chizhik S., Sidelnikov A. (2019). Synthesis and structural characterization of ceria nanoparticle agglomerates with shape inherited from an oxalate precursor. Ceram. Int..

[42] Zhao P., Song J., Xia M., Wang Y., Meng D., Wang H., Li R. (2017). Photocatalytic and electrochemical properties of CeO_2_ with lamellar structure inherited from cerium oxalate complex. J. Porous Mater..

[43] Brittain H.G., Sachs C.J., Lynch J.F., Ogle K.M., Perry D.L. (1987). Spectroscopic studies of the thermal decomposition products of hydrated cerous oxalate. Inorg. Chim. Acta.

[44] Millo A., Raichlin Y., Katzir A. (2005). Mid-infrared fiber-optic attenuated total reflection spectroscopy of the solid–liquid phase transition of water. Appl. Spectrosc..

[45] Nakamoto K. (2006). Infrared and Raman Spectra of Inorganic and Coordination Compounds. Handbook of Vibrational Spectroscopy.

[46] Li F., Koopal L., Tan W. (2018). Roles of different types of oxalate surface complexes in dissolution process of ferrihydrite aggregates. Sci. Rep..

[47] Andreescu D., Matijević E., Goia D.V. (2006). Formation of uniform colloidal ceria in polyol. Colloids Surf. A Physicochem. Eng. Asp..

[48] Darroudi M., Hoseini S.J., Oskuee R.K., Hosseini H.A., Gholami L., Gerayli S. (2014). Food-directed synthesis of cerium oxide nanoparticles and their neurotoxicity effects. Ceram. Int..

[49] Balakrishnan G., Raghavan C., Ghosh C., Divakar R., Mohandas E., Song J.I., Bae S., Kim T.G. (2013). X-ray diffraction, Raman and photoluminescence studies of nanocrystalline cerium oxide thin films. Ceram. Int..

[50] Jayakumar G., Irudayaraj A.A., Raj A.D. (2019). A comprehensive investigation on the properties of nanostructured cerium oxide. Opt. Quantum Electron..

[51] Khan M.M., Khan W., Ahamed M., Alhazaa A.N. (2017). Microstructural properties and enhanced photocatalytic performance of Zn doped CeO_2_ nanocrystals. Sci. Rep..

[52] Ansari S.A., Khan M.M., Ansari M.O., Kalathil S., Lee J., Cho M.H. (2014). Band gap engineering of CeO_2_ nanostructure using an electrochemically active biofilm for visible light applications. Rsc Adv..

[53] Choudhury B., Chetri P., Choudhury A. (2014). Oxygen defects and formation of Ce^3+^ affecting the photocatalytic performance of CeO_2_ nanoparticles. RSC Adv..

[54] Sharpe E., Andreescu D., Andreescu S. (2011). Artificial nanoparticle antioxidants. Oxidative Stress: Diagnostics, Prevention, and Therapy.

[55] Al-Attar A.M. (2011). Antioxidant effect of vitamin E treatment on some heavy metals-induced renal and testicular injuries in male mice. Saudi J. Biol. Sci..

[56] Collin V.C., Eymery F., Genty B., Rey P., Havaux M. (2008). Vitamin E is essential for the tolerance of Arabidopsis thaliana to metal-induced oxidative stress. Plant Cell Environ..

[57] Farias I.A.P., Santos C.C.L.D., Sampaio F.C. (2018). Antimicrobial activity of cerium oxide nanoparticles on opportunistic microorganisms: A systematic review. BioMed Res. Int..

[58] Thakur N., Manna P., Das J. (2019). Synthesis and biomedical applications of nanoceria, a redox active nanoparticle. J. Nanobiotechnology.

[59] Zhang M., Zhang C., Zhai X., Luo F., Du Y., Yan C. (2019). Antibacterial mechanism and activity of cerium oxide nanoparticles. Sci. China Mater..

[60] Zhang M., Yuan R., Chai Y., Wang C., Wu X. (2013). Cerium oxide–graphene as the matrix for cholesterol sensor. Anal. Biochem..

[61] Bellio P., Luzi C., Mancini A., Cracchiolo S., Passacantando M., Di Pietro L., Perilli M., Amicosante G., Santucci S., Celenza G. (2018). Cerium oxide nanoparticles as potential antibiotic adjuvant. Effects of CeO_2_ nanoparticles on bacterial outer membrane permeability. Biochim. Biophys. Acta (BBA) Biomembr..

[62] Sykes J.E., Sykes J.E. (2014). Chapter 36—Gram-negative Bacterial Infections. Canine and Feline Infectious Diseases.

[63] Hett E.C., Rubin E.J. (2008). Bacterial growth and cell division: A mycobacterial perspective. Microbiol. Mol. Biol. Rev..

