# Supplementary material for: Cerium Oxide Nanoparticles and Their Efficient Antibacterial Application In Vitro against Gram-Positive and Gram-Negative Pathogens
Source: Nanomaterials (Basel). 2020 Aug 18;10(8):1614. doi: 10.3390/nano10081614 (PMC7466638; doi:10.3390/nano10081614)
Supplement: Supplementary file 1 [file nanomaterials-10-01614-s001.pdf]

nanomaterials

Article

# Cerium Oxide Nanoparticles and Their Efficient Antibacterial Application in Vitro against Gram-Positive and Gram-Negative Pathogens

**Oana L. Pop [1], Amalia Mesaroş [2,*], Dan C. Vodnar [1], Ramona Suharoschi [1], Flaviu Tăbăran [1], Lidia Mageruşan [3], István Sz. Tódor [4], Zoriţa Diaconeasa [1], Adriana Balint [2], Lelia Ciontea [2] and Carmen Socaciu [1]**

1 Department of Food Science, University of Agricultural Science and Veterinary Medicine, 3-5 Calea Mănăştur Street, 400372 Cluj-Napoca, Romania; oana.pop@usamvcluj.ro (O.L.P.); dan.vodnar@usamvcluj.ro (D.C.V.); ramona.suharoschi@usamvcluj.ro (R.S.); flaviutabaran@gmail.com (F.T.); zorita.sconta@usamvcluj.ro (Z.D.); carmen.socaciu@usamvcluj.ro (C.S.)
2 Physics and Chemistry Department, C4S Centre, Technical University of Cluj-Napoca, 28 Memorandumului Street, 400114 Cluj-Napoca, Romania; balintadriana@yahoo.com (A.B.); Lelia.Ciontea@chem.utcluj.ro (L.C.)
3 National Institute for Research and Development of Isotopic and Molecular Technologies, 65-103 Donath Street, 400293 Cluj-Napoca, Romania; lidia.magerusan@itim-cj.ro
4 Faculty of Physics, Babeş-Bolyai University, 1st Kogălniceanu Street, 400084 Cluj-Napoca, Romania; istvan.todor88@gmail.com
* Correspondence: amalia.mesaros@chem.utcluj.ro

The formation of $Ce_2(C_2O_4)_3$ precursor - during the precipitation process of cerium nitrate and oxalic acid – is governed by the following chemical reaction:

$$2Ce(NO_3)_3 + 3H_2C_2O_4 \rightarrow Ce_2(C_2O_4)_3\downarrow + 6H^+ + 6NO_3^-$$

**Table S1.** The antioxidant activity data.

| $CeO_2$ np Concentration µM | µmol Trolox/g Sample | µmol Trolox/g Sample | µmol Trolox/g Sample |
|---|---|---|---|
| 5. | 184.1 | 184.6 | 184.2 |
| 10. | 193.0 | 194.0 | 192.9 |
| 25. | 320.9 | 320.9 | 321.0 |
| 50. | 401.9 | 401.7 | 401.5 |
| 75. | 451.9 | 451.7 | 452.0 |
| 150. | 478.0 | 477.8 | 479.1 |
| 250. | 482.8 | 482.6 | 481.6 |
